# A rare case of delayed splenic rupture following initial negative CT scan imaging: A case report and review of the literature

**DOI:** 10.1016/j.ijscr.2022.107517

**Published:** 2022-08-13

**Authors:** Margo Carlin, Adel Elkbuli, Piueti Maka, Mark McKenney, Dessy Boneva

**Affiliations:** a Department of Surgery, Division of Trauma and Surgical Critical Care, Kendall Regional Medical Center, Miami, FL, USA; b Department of Surgery, Division of Trauma and Surgical Critical Care, Orlando Regional Medical Center, Orlando, FL, USA; c Department of Surgical Education, Orlando Regional Medical Center, Orlando, FL, USA; d John A. Burns School of Medicine, Honolulu, HI, USA; e University of South Florida, Tampa, FL, USA

**Keywords:** Blunt abdominal trauma, Delayed splenic rupture, Hemorrhagic shock, Ultrasonography

## Abstract

**Introduction:**

Delayed splenic rupture is an often unpredictable event with high mortality. In this report, we discuss the successful management of delayed splenic rupture, presenting days after index injury, with no commonly associated injuries or blunt abdominal trauma.

**Case presentation:**

A 50 year old male, not on anticoagulants, presented with blunt trauma after driving his motorcycle into a tree. The patient sustained right 3–5 rib fractures, displaced right midclavicular fracture, 25 % right pneumothorax, T5–9 posterior spinous process fractures, left 2nd-5th metatarsal fractures, and scattered abrasions to the left foot, knee and hand. Focused abdominal sonography for trauma (FAST) and admission abdominal multi-detector CT were negative for any intra-abdominal injuries. On hospital day 5, the patient acutely decompensated. FAST was grossly positive and emergent laparotomy revealed a splenic rupture. After a splenectomy, he recovered.

**Discussion:**

The spleen is the most commonly injured organ in blunt abdominal trauma. Although acute injury often presents with imaging findings or sequelae of hemorrhagic shock, complications of splenic trauma have the potential to result in delayed catastrophe. Bedside ultrasonography is a useful tool to assess acute decompensation in trauma patients along with CT imaging. Prompt identification and hemorrhage control are crucial to survival after trauma.

**Conclusion:**

Repeat CT scans are also important for the identification of delayed splenic rupture and timely intervention. Delayed hemorrhage after blunt trauma should never be ruled out regardless of the injury complexity or length of hospital admission.

## Highlights

• A unique presentation of delayed splenic rupture after an initial negative work up
• Emphasizes the acute and delayed complications of acute splenic injuries
• Trauma patients who present after admission with shock need hemorrhage evaluation.
• FAST in hemodynamically unstable trauma patients can be potentially life-saving.

## 1 Introduction

One of the most commonly injured solid organs in blunt abdominal trauma is the spleen [1]. Anatomically, the spleen sits inferior to the left hemidiaphragm, lateral to the stomach, and superolateral to the colon. Although protected by the lower rib cage, left-sided rib fractures are associated with splenic trauma by direct laceration of the organ by the fractures or by the transmission of traumatic force.

The initial work-up of splenic trauma starts with a physical examination of the patient. The Focused Abdominal Sonography for Trauma (FAST) is a useful adjunct to identifying hemoperitoneum. However, a CT scan remains the gold standard for diagnosis in hemodynamically stable patients. Injury to the spleen may demonstrate contrast blush on arterial and delayed phases of the imaging study versus simple lacerations and capsular hematomas. Injury grading may be classified using the American Association for the Surgery of Trauma (AAST) grading system, the most widely used grading system for splenic trauma, based on imaging, operative, and pathologic findings.

Management of injury to the spleen is generally dependent upon the grade of injury and hemodynamics of the patient. Hemodynamically unstable patients warrant emergent exploratory laparotomy. However, there are multiple algorithms in the approach to the hemodynamically stable patient. Stable patients with a blush on CT are candidates for splenic preservation with angioembolization of the spleen. Patients with simple lacerations or hematomas can be managed conservatively with serial laboratory studies and abdominal exams. It should be kept in mind patients may fail at any point with life threatening hemorrhage and require operative intervention.

The spleen is crucial for the physiologic function of the reticuloendothelial system and essential for immunity. Splenic preservation is the most beneficial outcome to preserve immunologic function in the absence of hemodynamic instability. Preservation of the spleen can prevent overwhelming post-splenectomy infection (OPSI) which is associated with high mortality. Splenic injury grading scales are useful to help understand the risks of this injury complex [2]. Complications of the injury include acute and delayed hemorrhage, missed injuries, abscess formation, and overwhelming infection. Delayed splenic rupture (DSR) is a risk in the nonoperative management of splenic injuries and is associated with a higher mortality rate than acute injury (5–15 % versus 1 %) [3]. Here, we present a rare case of DSR after blunt trauma in the absence of solid organ injury on index imaging and presentation. This case has been reported in line with the SCARE criteria [4].

## 2 Case presentation

A 50-year-old male presented as a Level 1 activation, meeting physiologic/anatomic criteria necessitating an immediate full trauma team response. The patient presented with altered mental status, after driving his motorcycle into a tree. His vital signs were within normal limits and his initial Glasgow Coma Score (GCS) of 13 quickly improved. His past medical history revealed alcohol use disorder but no anticoagulants. He complained of right shoulder and back pain. The patient had a FAST that was negative and was stable enough to undergo chest and abdominal CT with IV contrast imaging studies, which revealed fractures of right ribs 3–5, right midclavicular fracture displacement, right pneumothorax, and T5–9 spinous process fractures. Both the FAST and CT imaging studies did not show any indications of splenic injury. The patient underwent a right tube thoracostomy with a resolution of the pneumothorax and was admitted to the trauma service for pulmonary optimization, control of pain, and treatment of his injuries.

On hospital day (HD) 3, the patient underwent placement of a right paravertebral subcutaneous pain pump for significant ongoing pain associated with his right-sided rib fractures. On HD-5, the trauma team was called to the bedside for acute hemodynamic compromise and the patient was found to be diaphoretic, hypotensive, tachycardic, and have altered mental status. He was intubated for inability to protect his airway and fluid resuscitation was initiated. The results of a stat bedside FAST examination revealed a large amount of fluid in the abdomen and a point of care hemoglobin was 5, down from 11, earlier in the day. A Massive Transfusion Protocol (MTP) was initiated, and the patient underwent an emergent laparotomy. In the operating theater, several liters of dark blood were evacuated from the abdomen and a hilar splenic injury was identified. The spleen was removed. Due to the patient being in extremis, damage control laparotomy was performed, and a negative pressure wound vacuum dressing was placed and ongoing resuscitation continued.

In the Trauma ICU, his blood pressure, fluid requirements, and pH stabilized over several hours while his urine output remained poor. On postoperative day one (POD-1) he was re-explored with no additional findings and a second negative pressure wound vacuum dressing was placed because of ongoing swelling and edema. On POD-4, the patient underwent a third laparotomy. No additional findings were encountered, the edema had improved, and after abdominal washout, the abdomen was closed. His course was complicated by acute kidney injury requiring hemodialysis. He was administered the post-splenectomy vaccines (Haemophilus b Polysaccharide Conjugated Vaccine, Meningococcal Polysaccharide Vaccine, and Pneumococcal 7-Valent Conjugated Vaccine) on POD-14. Over the next two weeks, his renal function improved, and he was discharged in a stable condition. A follow-up in the office two weeks later was unremarkable.

## 3 Discussion

Blunt trauma to the thorax, long bones or abdomen can cause dispersion of forces throughout the anatomy, leading to splenic injury. Thus, splenic injuries are the most common organ injury in blunt trauma. The overall management of this splenic injury accounts for hemodynamic status, specific anatomic injury, and other associated injuries as well as factors including initial imaging, patient age, institution experience, the individual surgeon, and trauma type [5]. Given the high vascularity of the spleen, bleeding is a likely consequence that can progress to life-threatening conditions. Therefore, the AAST Organ Injury Scale is useful in reflecting the severity of the injury as well as guiding management and aiding in prognosis. This case underscores that hemorrhage remains the most common etiology of hemodynamic instability in a trauma patient and that despite negative initial imaging on presentation and prolonged timing till hemorrhage event, a splenic injury is still a possibility.

Due to the major hematologic and immunologic role of the spleen, efforts are made to salvage the organ to the greatest extent. However, in cases of splenic rupture leading to ongoing shock, splenectomy is absolutely indicated, as in our patient's case [5]. The decision for a splenectomy following trauma has been difficult to determine, particularly in cases of hemodynamically stable patients with minimal signs of abdominal injury [5]. Current management of splenic injury in hemodynamically stable patients has highlighted nonoperative management (NOM). In known splenic injuries undergoing NOM, appropriate facilities and staff should be readily available [6]. In addition, angiography with angioembolization is a useful adjunct and preferred over exploratory laparotomy in a hemodynamically stable patient with concerning imaging findings [7]. However, higher grade injuries (i.e.: pseudoaneurysm [Grade 4] and hilar disruption [Grade 5]) represent increased risks for complications, which may demand intervention. Complications include delayed splenic ruptures, pseudocysts, pseudoaneurysms, and arteriovenous fistulas [8]. In cases where a splenic injury is unknown, the Western Trauma Association (WTA) notes that concern for missed injury should not influence initial decisions for NOM [6].

DSR is a complication that was first described in 1902 when Baudet presented a case 48 h after injury, defining the diagnosis [9]. There is no reliable method of predicting DSR, and the etiology of DSR is thought to arise from post-traumatic splenic lesions such as parenchymal pseudoaneurysms, subcapsular hematomas, splenic pseudocysts, or even rib fractures [3], [10]. The resulting splenic ruptures from blunt trauma can present as a classical triad with left hemidiaphragm elevation, left lower lobe atelectasis and left pleural effusion [10]. However, this triad is often absent and is not reliable [10]. Furthermore, previous literature has noted left-sided thoracic and abdominal injury dominance related to a splenic injury [11], [12]. Splenic injury should be ruled out, especially with thoracic injuries such as left-sided and bilateral rib fractures [3]. Yet, in our case, our patient presented with right-sided rib fractures, a right-sided pneumothorax, and even absent abdominal trauma. The presence of rib fractures suggests that high-force trauma has the potential to disrupt soft tissues, such as mesentery and solid organs. Thus, while splenic injury more commonly occurs with left-sided trauma, the risk of splenic damage is still possible and should always be considered even when injuries are contralateral or absent [11], [12].

DSR may also be due to missed injury from confounding medical history, lack of full work up, or imaging issues [13]. Blunt trauma to the abdomen can be missed in the initial clinical examination unless repeatedly searched for [14]. The lack of notable abdominal trauma findings prior to imaging in our case decreased the likelihood of splenic injury. Furthermore, miniscule splenic injuries may not be accurately identified in imaging studies leading to missed diagnoses [13]. The FAST exam is a useful clinical tool for imaging, especially in a hemodynamically unstable patient to identify hemoperitoneum at the bedside; however, the FAST is user-dependent and lacks the ability to identify sources of bleeding [1]. Additionally, up to 20 % of splenic trauma or peri-diaphragmatic injuries present without significant extravasation of blood, which may add to the increased possibility for false negative FAST exams [1]. Thus, CT scans are necessary to rule out associated splenic vascular injury. Intravenous contrast-enhanced CT scans are now the standard for diagnosing splenic injuries, with The EAST Practice and Management Guideline noting that CT scans are the most accurate, specific, and sensitive in identifying and characterizing splenic injury [15], [16]. CT scans often diagnose occult injuries and injuries that would have been missed if based on clinical examination solely; yet, the literature also suggests that the rate of missed splenic vascular injuries remains largely unknown [14], [17], [18]. Moreover, it is possible that index imaging may be inadequate and fail to capture vascular damage or parenchymal disruption, especially due to contrast timing or patient motion [13]. However, the finding of DSR after an initial negative work up using a multi-detector CT is 0.4 % of splenic injuries and probably lower if high quality scans are obtained [13]. Thus, the lack of positive findings suggesting splenic injury on both physical examination and imaging would discount the possibility of splenic injury including DSR, as in our case.

**Fig. 1:**
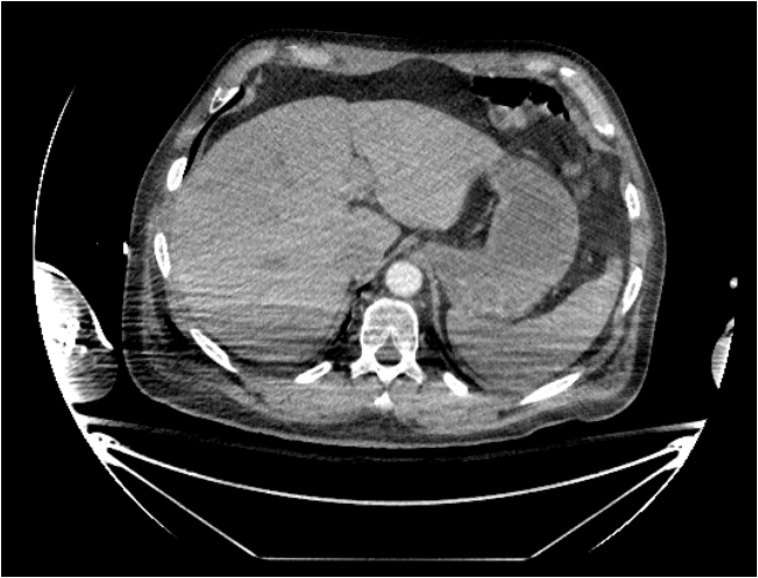
An axial view/cross-section of a CT scan of the abdomen reveals a normal appearance of the spleen with no evidence of splenic laceration on index imaging of the spleen.

DSR may therefore be countered by repeat imaging. There are currently no evidence-based algorithms recommending delayed imaging studies based on index radiography, which may contribute to the 3–15 % incidence of DSR [8]. Studies have found that repeat CT scans at 48 h of blunt splenic injury may be able to identify delayed splenic vascular trauma, splenic pseudoaneurysms (SPA), and/or arterial extravasation [17], [19], [20]. Furthermore, repeated CT scans have been recommended in patients with an AAST grade 2 splenic injury or higher, left lower posterior rib fractures, intraperitoneal fluid adjacent to the spleen, and/or injury of the left upper quadrant [17], [21]. The WTA has noted that recent data suggests specifically screening for SPA formation in splenic injuries via repeat CT scans due to their increasing incidence [6]. SPA formation and presence is an important risk factor for consideration of DSR, even in cases of minor splenic injuries [15]. Previous literature supports that routine CT follow-up identifies a significant proportion of patients with SPA; thus, repeated CT screening for SPAs may aid in identifying patients most at risk for DSR [23]. Furthermore, most delayed splenic vascular injuries have also been noted to be only detected on or are much more conspicuous on arterial phase CT scan imaging versus with portal-venous CT scan [17]. In our case, no definitive indications were present that splenic injury had occurred or should be suspected other than the trauma event itself, so delayed imaging was not obtained to screen for SPA and consequently DSR (Fig. 1).

For treatment in our patient, a splenectomy was ultimately completed to handle the DSR. Management of splenic injuries with NOM has become increasingly popular, even in more severely injured spleens [18]. NOM has had escalating success over the years, especially with the incorporation of splenic angioembolization as an adjunct [19], [21]. NOM has even shown to be effective in the management of DSR, with a previous study boasting an 83 % success rate in a small sample size [21]. However, lack of protocols, large variability in physician practice, and questionable clinical decision-making can contribute to the failure of NOM of splenic injuries [22]. Thus, in the presence of an established protocol, a stable patient, and an experienced physician, it is possible for DSR to be successfully managed nonoperatively.

This case reflects a very rare type of DSR with a complete absence of the clinical context consistent with delayed hemorrhages including negative associated traumatic injuries, blunt abdominal trauma, and radiographic findings. However, there have been similar reported cases in the literature reflecting a need for further classification of the risk factors [13]. Furthermore, this case emphasizes the management of complex blunt trauma with sudden deterioration, characteristic of critically ill trauma patients. The initial workup and management of splenic trauma should be reevaluated on a larger scale to fully understand the incidence of DSR.

## 4 Conclusion

This rare case of delayed splenic rupture emphasizes the management of complex blunt trauma with sudden deterioration, characteristic of critically ill trauma patients. The use of FAST and CT imaging should be used initially, with repeated CT imaging 48 h later to exclude abdominal injury. This case underscores that hemorrhage remains the most common etiology of hemodynamic instability in the trauma patient. Delayed hemorrhage after blunt trauma should never be ruled out regardless of the injury complexity or length of hospital admission.

## Funding

None.

## Ethical approval

This is a case report study. Informed patient written consent has been obtained and all identifying information was omitted.

## Consent

Informed patient written consent has been obtained and all identifying information is omitted.

## Author contribution

DB, MM, MC, AE – Conception of study, acquisition of data, analysis, and interpretation of data.

DB, MM, MC, AE – Drafting the article.

DB – Management of case.

DB, MM, MC, PM, AE – Critical revision of the article and final approval of the version to be submitted.

## Registration of research studies

Not applicable.

## Guarantor

Dessy Boneva.

Mark McKenney.

## Declaration of competing interest

None.
